# Difficulties in Getting to Sleep and their Association with Emotional and Behavioural Problems in Adolescents: Does the Sleeping Duration Influence this Association?

**DOI:** 10.3390/ijerph17051691

**Published:** 2020-03-05

**Authors:** Michaela Kosticova, Daniela Husarova, Zuzana Dankulincova

**Affiliations:** 1 Institute of Social Medicine and Medical Ethics, Faculty of Medicine, Comenius University in Bratislava, Sasinkova 2, 81372 Bratislava, Slovakia; 2 Department of Health Psychology and Research Methodology, Faculty of Medicine, P.J. Safarik University, Trieda SNP 1, 04011 Kosice, Slovakia; daniela.husarova@upjs.sk (D.H.); zuzana.dankulincova@upjs.sk (Z.D.)

**Keywords:** insufficient sleep duration, difficulties in getting to sleep, adolescents, emotional and behavioural problems

## Abstract

Sleep problems are common in adolescence with a negative impact on the mental health and functioning of adolescents. However, the roles of different sleep problems in relation to emotional and behavioural problems (EBPs), classified according to the 10th version of the International Classification of Diseases as emotional, conduct, hyperactivity and social functioning disorders, are not clear. The first aim of the study was to investigate the association between difficulties in getting to sleep and EBPs in adolescents. The second aim was to explore the role of sleep duration in this association. We used data from the Health Behaviour in School-aged Children (HBSC) study conducted in 2018 in Slovakia. Presented are results for specific age groups of 13-year-old (N = 1909) and 15-year-old (N = 1293) adolescents. Subjective measures of sleep variables were used. Binary logistic regression models adjusted for age and gender were used to assess associations between difficulties in getting to sleep, sleep duration and EBPs measured using the Strengths and Difficulties Questionnaire. Modification of the association between difficulties in getting to sleep and EBPs by sleep duration was also explored. We found that difficulties in getting to sleep at least once a week as well as insufficient sleep (less than 8 h) increased the probability of EBPs. Interactions of sleep duration with difficulties in getting to sleep on EBPs were found to be non-significant. The results suggest that caregivers and clinicians should screen and intervene for both sleep quality and quantity problems in adolescents as they might indicate and promote EBPs.

## 1. Introduction

Emotional and behavioural problems (EBPs) in adolescents refer to the group of disorders classified according to the 10th revision of International Classification of Diseases as “Behavioural and emotional disorders with onset usually occurring in childhood and adolescence” (F90-F98) including hyperkinetic disorders, conduct disorders, emotional disorders, disorders of social functioning and tic disorders [1]. EBPs which are common in adolescence with prevalence of 10%–25% can have long-lasting consequences not only for adolescents but also for their families and society as a whole [2,3]. Optimal sleep quantity and quality is essential for the mental health, adequate functioning and well-being of adolescents [4,5]. However, 30% to 50% of adolescents sleep less than recommended [6,7,8,9] and the prevalence of sleep difficulties, manifested as difficulties in falling or staying asleep, range from 10% to 20% in adolescents [10,11,12,13,14,15]. According to the recommendations of American Academy of Sleep Medicine (AASM), adolescents of 13 to 18 years old should sleep 8 to 10 h per 24 h on a regular basis to promote optimal health [16]. As sleep quality is a multidimensional construct, the recommendations regarding good sleep quality are not so clear. Experts from the National Sleep Foundation have reached a consensus only for some indicators of good sleep quality in adolescence including sleep onset latency of less than 30 minutes and maximum one awakening (>5 mins) per night [5]. The results of the recent studies indicate an increasing trend in the prevalence of sleep difficulties [13,17] and consistent decline in sleep duration over time and across adolescence [18,19,20]. 

Both insufficient sleep duration and poor sleep quality constitute risk factors for adolescents’ neurodevelopment with significant long-term consequences on mental health and functioning [21,22,23]. Inadequate sleep contributes to problems with emotional and behavioural regulation and increases the risk for psychopathology [24,25]. Poor sleep is associated with various emotional, cognitive and behavioural problems including depression, anxiety, attention-deficit hyperactivity disorder, risk-taking behaviour, aggression, suicidality and poor academic performance [4,24,26]. Moreover, persistent sleep difficulties occurring at least 3 nights/week and for at least 3 months are classified as insomnia disorders [27,28] and are considered not only a risk factor for the development of certain mental conditions but also a potential warning sign for serious mental disorders [15,29]. 

Evidence suggests that sleep duration and sleep quality represent two separate sleep domains [30] and their association with EBPs is bidirectional and complex [23,31]. However, it remains unclear what kind of roles sleep duration and sleep quality play in this association and how they influence each other. Therefore, the aims of the present study were (1) to investigate the association between difficulties in getting to sleep and EBPs and (2) to explore the role of sleep duration on this association.

## 2. Materials and Methods 

### 2.1. Sample and Procedure

We used data from the Health Behaviour in School-aged Children (HBSC) study conducted in 2018 in Slovakia, which is part of World Health Organisation collaborative, cross-national HBSC study of 11-, 13- and 15-year-old adolescents from 49 countries and regions across Europe and North America [32]. The HBSC used two-step sampling to obtain a representative sample. In the first step, 140 larger and smaller elementary schools located in rural as well as in urban areas from all regions in Slovakia were asked to participate. These were randomly selected from a list of all eligible schools in Slovakia obtained from the Slovak Institute of Information and Prognosis for Education. The school response rate (RR) was 77.9%. In the second step, we obtained data from 8405 adolescents from the fifth to ninth grades of the elementary schools in Slovakia in the target group of 11 to 15 years old (mean age 13.43; 50.9% boys); one class per grade was selected. Presented are results for specific age groups of 13-year-old (N = 1909) and 15-year-old (N = 1293) adolescents (mean age 13.81; 53% boys). 

### 2.2. Ethics

The study was approved by the Ethics Committee of the Medical Faculty at P.J. Safarik University in Kosice (16N/2017). Parents were informed about the study via the school administration and could opt out if they disagreed with their child’s participation. Participation in the study was fully voluntary and anonymous with no explicit incentives provided for participation. 

### 2.3. Measures

Difficulties in getting to sleep were measured using one question (symptom) from the HBSC-symptoms checklist (HBSC-SCL), which assessed the occurrence of eight subjective psychosomatic health complaints. The response categories indicating how frequently during the last 6 months the symptoms had occurred are "rarely or never", "about every month", "about every week", "more than once a week" and "about every day". Responses for specific health complaints were dichotomized ("rarely or never" and "about every month" vs. "about every week", "more than once a week" and "about every day") [33]. 

Sleep duration during school days was calculated by estimating the time between bedtime and rise time. Bedtime was measured with the item: “At what time do you usually go to bed, when you are going to school the morning after?” Rise time was measured with the item: “At what time do you usually get up, when you are going to school?” According to the recommendations of the AASM [16] 13- and 15-year-old adolescents should sleep 8–10 h regularly. Sleep durations less than 8 h were coded as “insufficient”, sleep durations 8 h and more as “sufficient”. 

Emotional and behavioural problems were measured with the Strengths and Difficulties Questionnaire (SDQ), which includes 25 items on psychological attributes [34,35], of which we used the 20 problem items divided between four subscales of five items each: emotional problems (somatic, worries, unhappy, clingy, fears; e.g., “I have many fears, I am easily scared”), peer problems (solitary, good friend, popular, bullied, best with adults; e.g., “I have one good friend or more”), conduct problems (tempers, obedient, fights, lies, steals; e.g., “I am often accused of lying or cheating”) and hyperactivity problems (restless, fidgety, distractible, reflective, persistent; e.g., “I am restless, I cannot stay still for long”). Response categories were not true (0), somewhat true (1), certainly true (2). The sum scores of these 20 items range from 0 to 40, with a higher score indicating more problems. We dichotomised the SDQ scores into two categories—adolescents having problems (“abnormal”, score 20–40) and adolescents having no problems (“normal” and “borderline”, score 0–19).

### 2.4. Statistical Analysis

First, we described the sample using descriptive statistics. Second, we assessed the associations of the difficulties in getting to sleep with EBPs using binary logistic regression analysis (Model 1). Third, in order to assess the role of sleep duration as a mediator, we added sleep duration to the binary logistic regression in Model 2. Finally, we explored the modification of the associations of difficulties in getting to sleep with EBPs by sleep duration (Model 3). All three models were adjusted for age and gender. Statistical analyses were performed using SPSS v.20.

## 3. Results

### 3.1. Baseline Characteristics

Table 1 shows the basic descriptive statistics of the studied variables stratified by age and gender. Difficulties in getting to sleep were reported by 17% of boys and 30% of girls in both age groups. Most of the 13-years-old adolescents slept for the recommended 8 h or more, while only half of the 15-years-old adolescents met this recommendation. Almost 20% of 15-year-olds and more than 16% of 13-year-old girls had an abnormal SDQ score indicating EBPs, while in boys the prevalence was only 12% and 10% (Table 1). 

### 3.2. Associations of Difficulties in Getting to Sleep, Sleep Duration and EBPs

Table 2 presents the associations from binary logistic regression models of difficulties in getting to sleep and sleep duration with EBPs adjusted for age and gender. We found that the girls were more likely to have EBPs than boys (OR: 1.37, CI: 1.1−1.74). The results of Model 1 show that difficulties in getting to sleep were significantly associated with EBPs, with difficulties at least once a week increasing the probability of EBPs more than three times (OR: 3.3, CI: 2.61−4.16). In the next step we added sleep duration to the model (Model 2), and even though difficulties in getting to sleep remain significant, the presented odds ratios decreased slightly suggesting potential mediation (OR: 3.08, CI: 2.43−3.90). We also found sleep duration to be significantly associated with EBPs, with insufficient sleep less than 8 h increasing the probability of EBPs (OR: 1.56, CI: 1.24−2.00); however, the association was less strong than for difficulties in getting to sleep. Finally, we tested interactions of sleep duration with difficulties in getting to sleep on EBPs, but we found them to be non-significant (OR: 0.94, CI 0.59−1.50), suggesting independent associations with EBPs. 

## 4. Discussion

We found difficulties in getting to sleep to be significantly associated with EBPs in adolescents, with difficulties at least once a week increasing the probability of EBPs. Sleep duration seems to mediate but not moderate this association. Our results have confirmed the presumption that sleep duration and sleep quality are two separate sleep domains which are independently associated with EBPs in adolescents, with stronger association for sleep onset difficulties than for insufficient sleep duration. However, our previous research found that more adolescents had problems with lack of sleep than with falling asleep. Furthermore, the amount of sleep decreased with age and the problems with falling asleep and EBPs were more common in girls than boys [8,36].

Associations between poor sleep quantity and quality and EBPs have been confirmed by several studies. Adolescents with insufficient sleep duration (less than 8 h) and difficulties in getting to or maintaining sleep are at the higher risk of having symptoms of depression and anxiety [37,38,39], suicidal ideation [11,40,41,42], hyperactivity-inattention problems [43] and engaging in risky [25], violent and aggressive behaviour [44,45]. 

The evidence from experimental studies suggests that these associations might be causal and bidirectional. Adolescents who were exposed to sleep restrictions, even for one night, had worsened mood and decreased ability to control negative emotion and thus greater vulnerability to EBPs [46,47,48]. The study of Brand et al. [49] found that adolescents with higher mental toughness had objectively and subjectively better sleep quality and this association was likely bidirectional. It has been discussed in several studies that adverse changes in emotional regulation might be one pathway by which sleep loss and sleep onset difficulties increase the risk of EBPs [6,12,44,50]. The underlying mechanism is related to the changes in brain functions and structures with several hypotheses explaining how insufficient sleep deteriorates optimal neurodevelopment and functioning [51,52,53].

In our study, we found that poor sleep quality had stronger association with adverse mental and behavioural outcomes than insufficient sleep duration. The findings are consistent with the results of other studies [30,54,55,56]. Smaller effects of sleep duration can be explained by inter-individual variability in sleep needs and different individual vulnerability to sleep loss; thus, sleeping less than recommended time is not necessarily associated with adverse outcomes [4,6,16,30]. Moreover, most adolescents voluntarily choose a late bedtime because they prefer rewarding activities to going to bed earlier, however, those who have difficulties in getting to sleep are involuntarily awake and worrying about their sleep, which leads to distress and higher risk of EBPs [55]. Furthermore, there is evidence that sleep quantity and quality have different impacts on the mental health of adolescents and are related to specific emotional and mental health problems [43,57]. We found that sleep onset difficulties and EBPs are more common in girls compared to boys, which might suggest, based on the results of other studies, that girls are more vulnerable to the disruptive effects of sleep problems and vice versa [11,55].

### 4.1. Strengths and Limitations

The strengths of the study include a large representative sample, the use of valid HBSC and SDQ questionnaires and empirically based sleep recommendations.

There are some limitations in the study. First, the cross-sectional design does not allow causal relationships to be established. We are uncertain whether the insufficient sleep and difficulties in getting to sleep are determinants or outcomes of EBPs. Second, we used subjective measures of sleep quality and duration, which are not able to measure sleep as precisely as objective measures, such as actigraphy. Nevertheless, this is common in questionnaire studies that cover a broad range of topics and a large sample of participants, such as the HBSC study. Third, the sleep duration was calculated as a difference between wake-up time and bedtime, which is the time spent in bed and not the time spent asleep. This could have caused an overestimation of the number of sufficient sleepers. Finally, we used just one indicator of sleep quality—difficulties in getting to sleep—and did not measure the other domains of sleep quality such as problems with maintaining sleep and awakening difficulties, thus the findings cannot be applied to overall sleep quality. 

### 4.2. Implications

The results of the study suggest that clinicians and caregivers should screen for sleep problems as indicators of EBPs in adolescents and focus both on insufficient sleep duration and sleep quality problems. To improve the emotional well-being and functioning of adolescents, multilevel interventions to promote healthy sleep are needed. There is strong evidence that cognitive-behavioural interventions on an individual level and delaying the school start time on a population level can be effective methods to improve sleep and mental health outcomes in adolescence [58,59,60,61]. Development of interventions focusing on limitation of media device use during bedtime seems also be important to promote healthy sleep in adolescents [62,63,64]. In order to develop more effective interventions, higher quality research examining the causal pathways between sleep problems and EBPs and factors influencing this relationship is needed. Moreover, the results of this study emphasize the importance of combining different types of sleep quality measures and the use of objective sleep measures in future research. Finally, the national HBSC study is part of the international HBSC study running in more than 40 countries every 4 years, so the results could be used to compare the data between the countries and analyze time trends in future research. 

## 5. Conclusions

We found that difficulties in getting to sleep and insufficient sleep duration are two separate sleep domains independently associated with mental and behavioural problems in adolescents with stronger association for sleep onset difficulties. Despite some limitations of the study, our findings suggest that caregivers and clinicians should screen for both sleep quality and quantity problems in adolescents as they might indicate EBPs. In order to promote the mental health and well-being of adolescents, multilevel interventions to promote regular, optimal and healthy sleep are needed.

## Figures and Tables

**Table 1:** Descriptive statistics of studied variables stratified by age and gender (Slovakia, 2018, 13- and 15-year-olds).

Studied Variables	13 Years Old N = 1909		15 Years Old N = 1293	
	Boys	Girls	Boys	Girls
**Difficulties in Getting to Sleep**				
at least once a week (N)	158	234	111	173
at least once a week (%)	17.1%	27.1%	17.0%	31.2%
**Sleep duration**				
sufficient sleep duration (8 h and more) (N)	675	597	363	289
sufficient sleep duration (8 h and more) (%)	70.2%	65.5%	53.1%	49.7%
**EBPs**				
abnormal SDQ score (N)	102	127	63	100
abnormal SDQ score (%)	12.1%	16.2%	10.4%	19.6%

**Table 2:** The association of difficulties getting to sleep with emotional and behavioural problems and the moderation by sleep duration adjusted for age and gender from binary logistic regression (odds ratios—OR, 95% confidence intervals—95%CI) (Slovakia, 2018, 13- and 15-year-olds).

Independent Variables	EBPs		
	Model 1 OR, 95%CI	Model 2 OR, 95%CI	Model 3 OR, 95%CI
**Age**			
13 years old 15 years old	Ref. 0.99 0.79|1.24	Ref. 0.91 0.72|1.15	Ref. 0.91 0.72|1.15
**Gender**			
boys girls	Ref. 1.39 1.10|1.74 **	Ref. 1.37 1.09|1.72 **	Ref. 1.37 1.09|1.72 **
**Difficulties in getting to sleep**			
less than once a week at least once a week	Ref. 3.30 2.61|4.16 ***	Ref. 3.08 2.43|3.90 ***	Ref. 3.19 2.27|4.47 ***
**Sleep duration**			
sufficient (8 h and more) insufficient (less than 8 h)		Ref. 1.56 1.24|2.00 ***	Ref. 1.60 1.20|2.14 **
Difficulties in getting to sleep * Sleep duration			
less than once a week *sufficient at least once a week *insufficient			Ref. 0.94 0.59|1.50

* *p* < 0.05 ** *p* < 0.01 *** *p* < 0.001.
